# Romidepsin (FK228) regulates the expression of the immune checkpoint ligand PD-L1 and suppresses cellular immune functions in colon cancer

**DOI:** 10.1007/s00262-020-02653-1

**Published:** 2020-07-06

**Authors:** Yehui Shi, Ying Fu, Xin Zhang, Gang Zhao, Yuan Yao, Yan Guo, Gang Ma, Shuai Bai, Hui Li

**Affiliations:** 1 Medical Oncology Department of Breast Cancer, Tianjin Medical University Cancer Institute and Hospital, Huanhuxi Road, Hexi District, Tianjin, 300060 People’s Republic of China; 2 Phase I Clinical Trial Ward, Tianjin Medical University Cancer Institute and Hospital, Tianjin, 300060 People’s Republic of China; 3 Department of Gastrointestinal Cancer Biology, Tianjin Medical University Cancer Institute and Hospital, Tianjin, 300060 People’s Republic of China; 4 National Clinical Research Center for Cancer, Tianjin, 300060 People’s Republic of China; 5 Key Laboratory of Cancer Immunology and Biotherapy, Tianjin, China, Tianjin, 300060 People’s Republic of China; 6 Department of Cancer Biobank, Tianjin Medical University Cancer Institute and Hospital, Tianjin, 300060 People’s Republic of China

**Keywords:** Histone deacetylase inhibitor (HDACi), Romidepsin, PD-L1, BRD4, Regulatory T cells, Colon cancer

## Abstract

Romidepsin (FK228), a histone deacetylase inhibitor (HDACi), has anti-tumor effects against several types of solid tumors. Studies have suggested that HDACi could upregulate PD-L1 expression in tumor cells and change the state of anti-tumor immune responses in vivo. However, the influence of enhanced PD-L1 expression in tumor cells induced by romidepsin on anti-tumor immune responses is still under debate. So, the purpose of this study was to explore the anti-tumor effects and influence on immune responses of romidepsin in colon cancer. The results indicated that romidepsin inhibited proliferation, induced G0/G1 cell cycle arrest and increased apoptosis in CT26 and MC38 cells. Romidepsin treatment increased PD-L1 expression in vivo and in vitro via increasing the acetylation levels of histones H3 and H4 and regulating the transcription factor BRD4. In subcutaneous transplant tumor mice and colitis-associated cancer (CAC) mice, romidepsin increased the percentage of FOXP3+ regulatory T cells (Tregs), decreased the ratio of Th1/Th2 cells and the percentage of IFN-γ+ CD8+ T cells in the peripheral blood and the tumor microenvironment. Upon combination with an anti-PD-1 antibody, the anti-tumor effects of romidepsin were enhanced and the influence on CD4+ and CD8+ T cells was partially reversed. Therefore, the combination of romidepsin and anti-PD-1 immunotherapy provides a more potential treatment for colon cancer.

## Introduction

Histone deacetylases (HDACs) are important mediators of epigenetic regulation by removing acetyl groups from lysine residues of histones and some nonhistone proteins. Deacetylation of histones can affect chromatin conformation and inhibit gene expression,and the stability and biological functions of some transcription factors are also influenced by their acetylation state. HDACs are phylogenetically classified as class I (HDACs 1, 2,3 and 8), class II (HDACs 4–7, 9 and 10), class III (SIRTs 1–7)s and class IV (HDAC 11). Dysfunction of HDACs has been confirmed to be closely related to the occurrence and development of tumors [1].

HDAC inhibitors (HDACis) can inhibit HDAC-mediated deacetylation, causing the hyperacetylation of histones and the re-expression of epigenetic silencing genes [2]. Importantly, HDACis exert anti-tumor effects through varieties of pathways, including inducing cell cycle arrest (p21, cyclins) and cell apoptosis, regulating cell autophagy, inhibiting tumor angiogenesis (HIF-1a, VEGF), as well as regulating immune response (antigen presentation, T cell activation and Tregs differentiation) [3–8]. The first HDAC inhibitor vorinostat (also known as Zolinza or SAHA) was approved by US FDA for the treatment of cutaneous T cell lymphoma in 2006. Thereafter, romidepsin (FK228), belinostat (PXD101) and panobinostat (LBH589) were successively approved by FDA for the treatment of cutaneous T cell lymphoma (CTCL), peripheral T-cell lymphoma (PTCL) and multiple myeloma (MM). HDACis have become a hot spot in anti-tumor drug research.

Romidepsin, a naturally occurring selective inhibitor of HDACs 1 and 2, has been reported to induce cell cycle arrest and apoptosis in various solid tumor cells, including ovarian cancer and hepatocellular carcinoma [9, 10]. In addition to direct cytotoxicity, romidepsin can cause a wide range of immune changes like other HDAC inhibitors through the expression of costimulatory molecules (PD-L1), MHC, tumor antigens and cytokines [11–14]. It has been reported that HDACi increased the expression of FOXP3 and the suppressive activity of regulatory T cells in inflammation and transplantation models [15–17].

Therefore, we analyzed the efficacy of romidepsin in upregulating PD-L1 in murine colon cancer cells and its influence on T cell functions in this study. Although the role of PD-L1 upregulation in immune response against tumor has been controversial, the results herein demonstrated that the combination of romidepsin and anti-PD-1 treatment could effectively inhibit tumor growth in vivo. These data provide a potential option for combinatorial therapy for treating colon cancer.

## Materials and methods

### Cell lines and reagents

The mouse colon cancer cell lines CT26 and MC38 were obtained from the ATCC (Manassas, VA, USA) and cultured in RPMI-1640 with 10% FBS. All cells were maintained under humidified condition (37 °C, 5% CO_2_), and continual culture did not exceed 2 months. Romidepsin was purchased from Selleck Chemicals (Houston, TX, USA). An anti-PD-1 antibody was purchased from Biolegend (San Diego, CA, USA). Nontarget and BRD4-targeting siRNAs were purchased from RiboBio Co., Ltd. (Guangdong, China).

### Antibodies

The following antibodies (Abs) were used for Western blotting: rabbit anti-pan-Akt mAb (#4691), anti-p-Akt (T308) mAb (#4056), anti-p-Akt (S473) mAb (#4060), anti-pan-ERK mAb (#4695), anti-p-ERK (T202/Y204) mAb (#4376), anti-BRD4 (#13,440), anti-H3(#4499), anti-H4(#13,919), anti-acetyl-H3(Lys9/Lys14) (#9677), anti-acetyl-H3 (Lys27) (#8173), anti-acetyl-H4 (Lys5) (#8647), anti-acetyl-H4 (Lys8) (#2594), anti-acetyl-H4 (Lys16) (#13,534) and anti-acetylated-lysine (#9441); all of these were purchased from Cell Signaling Technology (Danvers, MA, USA). Mouse anti-GAPDH mAb (G8795) was purchased from Sigma-Aldrich. Goat anti-PD-L1 (AF1019) was purchased from R&D Systems (Minneapolis, MN, USA).

### Apoptosis and proliferation analysis using flow cytometry

Apoptosis and proliferation in CT26 and MC38 cells were analyzed using an Annexin V-FITC Apoptosis Detection Kit II (BD) and BrdU Cell Proliferation Assay Kit (BD), respectively. The results were measured using a FACS Canto II (BD). Representative results of independent assays are shown.

### RNA interference

For the transient knockdown of BRD4 expression, CT26 and MC38 cells were seeded in 6-well plates and transfected with nontarget or BRD4-targeting siRNA pool using HiPerFect (Qiagen, German) according to the manufacturer's instructions. The working concentration of siRNAs was 100 nM.

### RNA and RT-qPCR

RNA was extracted using TRIzol (Thermo Fisher Scientific), and cDNA was synthesized using a PrimeScript™ RT Reagent Kit (TaKaRa). The qPCR assays were performed using SYBR® Premix Ex Taq™ II (TaKaRa) and a QuantStudio™ 5 Real-Time PCR System (Thermo Fisher Scientific). GAPDH was used simultaneously as the internal control. One representative result of three independent assays is shown.

### Western blotting

Cell lysis buffer (100 mM NaCl, 10 mM EDTA (pH 8.0), 50 mM Tris–Cl (pH 8.0) and 0.5% (v/v) Triton X-100) with EDTA-free complete protease and phosphatase inhibitors (Roche) was used for protein extraction. The lysates were separated on a 10% SDS-PAGE gel and transferred onto PVDF membranes. The targets were detected using an Amersham Imager 600 (GE). GAPDH was used as the loading control.

### Immunoprecipitation

The CT26 and MC38 cells were treated with romidepsin for 24 h. Then, a Universal Magnetic Co-IP Kit (Active Motif) was used to harvest the acetylated proteins. Western blotting was used to detect the levels of histone 3 and histone 4 in the acetylated proteins.

### Subcutaneous transplantation tumor models

This study was carried out in accordance with laboratory animal-guideline for Ethical review of animal Welfare and approved by Institutional Animal Care and Use Committee of Tianjin Medical University Cancer Institute & Hospital (approval No. 2014035). Six-week-old female BALB/C mice (Beijing Vital River Laboratory Animal Technology Co., Ltd) were acclimatized in the Laboratory Animal Resource Center at Tianjin Tumor Hospital. CT26 cells (1 × 10^6^) diluted in PBS were implanted subcutaneously (s.c.) in the flank regions of the mice. When the tumors became palpable, 32 mice were randomized into four experimental groups. Based on previous studies, the mice were given intraperitoneal (i.p.) injections of 300 μg per mouse anti-PD-1 (diluted in sterile PBS) on days 14, 16,18,20,22,24,26,28,30 post-tumor implantation and romidepsin (1 mg/kg dissolved in DMSO and diluted in sterile PBS) on days 15, 17,19,21,23,25,27,29. This schedule was repeated for a total of 30 days. The mice in the control group were treated with PBS. The mice were killed when the tumors reached a length ≥ 2 cm. Tumor volumes (mm^3^) were calculated with the following formula: tumor volume = 1/2 (length × width^2^), where the width and the length were the shortest and the longest diameters, respectively, as measured by calipers every 2 days. At the end of the study, the mice were killed. The tumors were weighed and prepared as lysates.

### Colitis-associated cancer (CAC) models

CAC was induced with azoxymethane (AOM)/dextran sodium sulfate (DSS) as follows: Briefly, C57BL/6 mice were injected intraperitoneally with 12.5 μg/g AOM (Sigma, St. Louis, MO, USA) on the first day. After 5 days, the mice were given drinking water containing 3% DSS for 5 days. On day 70, 28 mice were randomized into four experimental groups (*n* = 7). Based on previous studies, the mice received i.p. injections of 300 μg per mouse anti-PD-1 (diluted in sterile PBS) on days 72, 74 and 76 and romidepsin (1 mg/kg dissolved in DMSO and diluted in sterile PBS) on days 71, 73 and 75. The colons were collected, and the tumors were counted and measured.

### Flow cytometry analysis

The following mouse Abs were used for flow cytometry analyses: anti-CD25 (101,908), anti-CD8 (100,706), anti-IFN-γ (113,604) and anti-PD-L1 (124,311); these antibodies were all purchased from Biolegend. Anti-CD4 (# 45–0042-82), anti-FOXP3 (#17–5773-82) and anti-IL-4 (# 25–7041-82) were purchased from Thermo Fisher. Gates were determined using isotype control Ab staining. Data were acquired with a FACS Canto II (BD) and expressed as the mean of five separate experiments.

### Statistical analysis

The data were analyzed by two-tailed Student’s *t* test or ANOVA using GraphPad Prism software (La Jolla, CA). Comparisons between groups are presented as the mean ± SEM. Values of *p* < 0.05 were considered statistically significant.

## Results

### Romidepsin showed anti-tumor activity against murine colon cancer

**Fig. 1:**
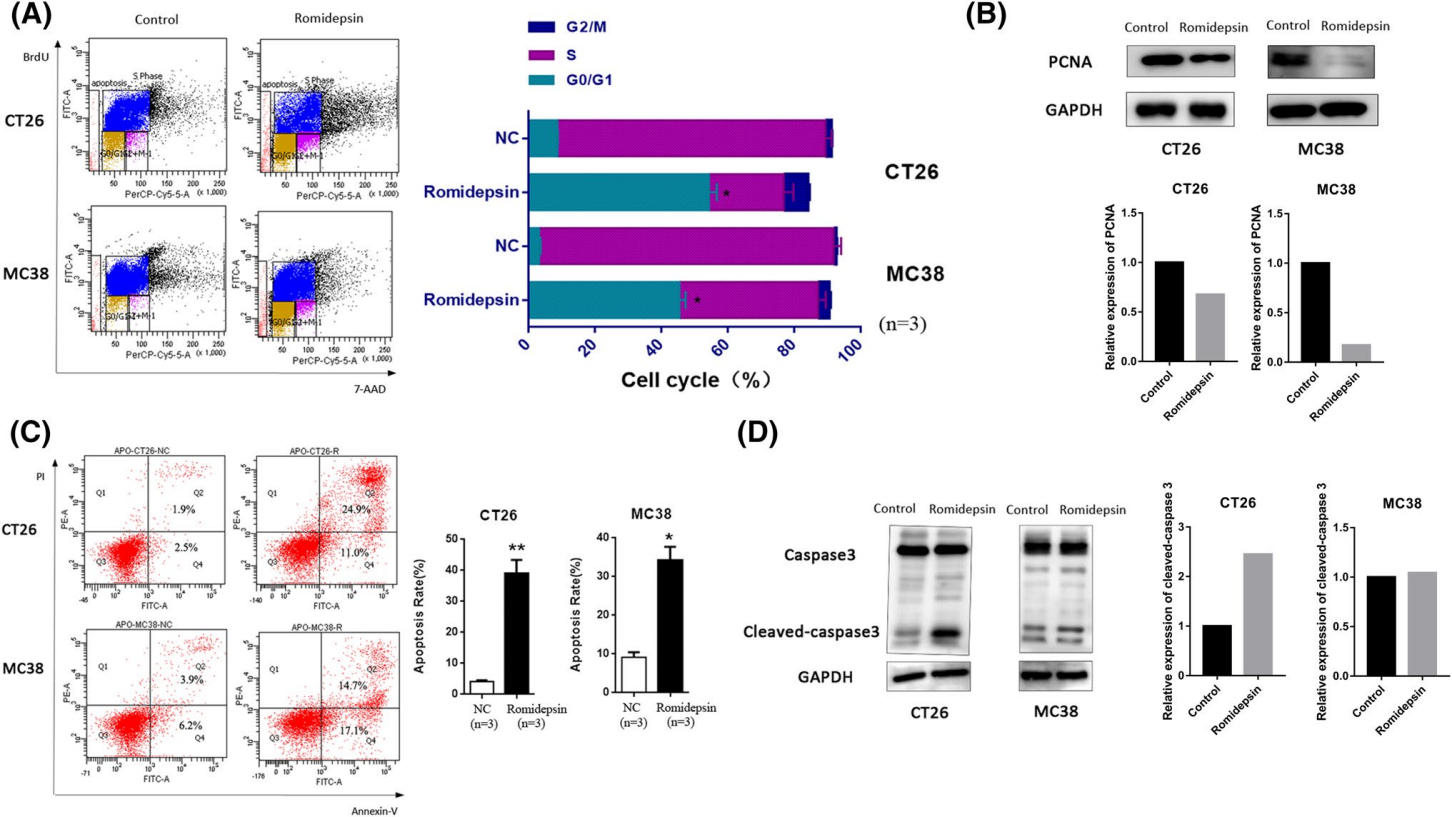
Romidepsin inhibited proliferation, induced cell cycle arrest and increased apoptosis in CT26 and MC38 cells. **a** The effects of romidepsin on the cell cycle were evaluated in murine colon cancer cell lines. CT26 and MC38 cells were treated with or without romidepsin (10 μM for CT26 cells and 40 nM for MC38 cells) for 24 h and then cultured with Brdu (10 ul/mL) for 4 h. The cell cycle profile was measured by flow cytometry to detect the incorporation of Brdu into DNA during cell proliferation. The bar chart displays the percentages of cells in each phase. Three replicates (*n* = 3) were set for each experimental group. **b** Expression levels of PCNA were determined by western blot with anti-PCNA antibodies. The histogram was used to analyze the fold change of sample grayscale from western blot result. **c** Apoptosis was examined using an Annexin V/PI assay. The cells in the region of Q2 and Q4 were defined as apoptotic cells. Three replicates (*n* = 3) were set for each experimental group. **d** The level of caspase-3 was determined by Western blot. The histogram was used to analyze the fold change of sample grayscale from western blot result (*P < 0.05, **P < 0.01)

**Fig. 2:**
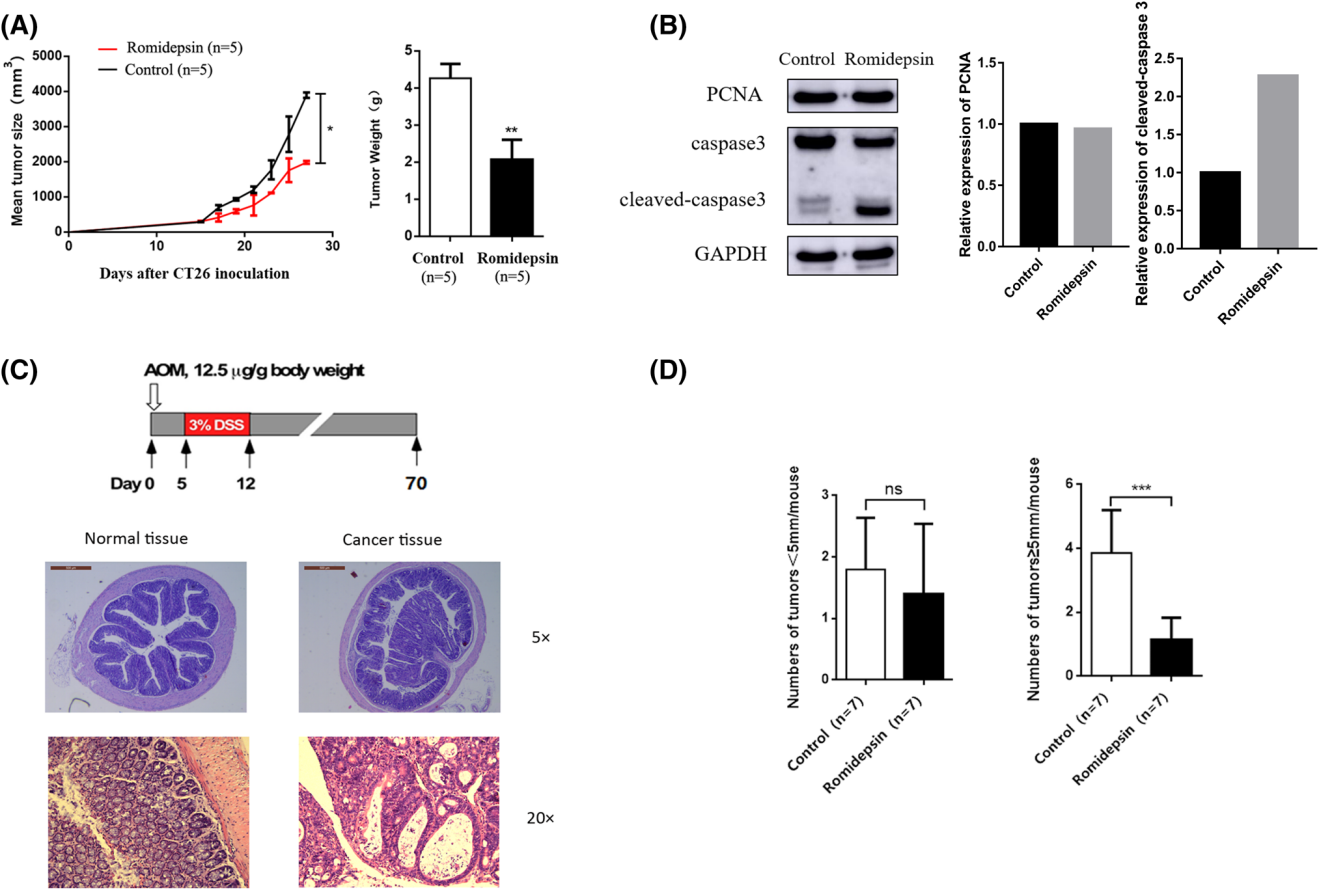
Romidepsin inhibited the growth of murine colon cancer in vivo*.* **a** Subcutaneous transplantation tumor mouse models were established by inoculating CT26 cells. A total of 10 mice were divided into two groups. When the subcutaneous transplanted tumors were visible on day 15, the experimental group (*n* = 5) was treated with romidepsin (1 mg/kg) every 2 days while the control group (*n* = 5) was injected with saline. Tumors were collected and weighed on day 30. The left line graph reflects the change of tumor size after treatment with or without romidepsin. The right histogram reflects the final difference of tumor weight between different groups. **b** Western blot was used to analyze the expression levels of PCNA and caspase 3 in control and romidepsin group. **c** In colitis-associated cancer (CAC) models, mice were treated with AOM (12.5 μg/g body weight) by intraperitoneal injection on the first day and 3% DSS in their drinking water from day 5 to day 12. The mice were continuously fed until killing on the 70th day, and the colon tumor was paraffin-embedded and stained with H&E. **d** Fourteen mice with colitis-associated cancer were randomly divided into two groups, and each group was treated with or without romidepsin (1 mg/kg, on days 71, 73 and 75). The average number of tumors per mouse was divided into two groups based on size (< 5 mm and ≥ 5 mm in the largest dimension) (*P < 0.05, **P < 0.01, ***P < 0.001)

The effects of romidepsin on the proliferation, cell cycle and apoptosis of the murine colon cancer cell lines were evaluated. CT26 and MC38 cells were treated with or without romidepsin for 24 h, then measured by Brdu cell cycle assay. We observed a dramatic decrease in the number of cells in the S + G2/M phase and a significant increase in the number of cells in the G0/G1 phase after romidepsin treatment (Fig. 1a). And the expression level of PCNA was also downregulated, which suggested the proliferation inhibition effects of romidepsin (Fig. 1b). Compared to the control cells, romidepsin-treated cells had a marginal increase in apoptotic events (Fig. 1c), which was validated by the increased caspase 3 cleavage (Fig. 1d). We further examined the responses of romidepsin in vivo using subcutaneous tumor-transplanted mice and CAC mice. Subcutaneous transplantation tumor mouse models were established by inoculating CT26 cells. After 16 days of treatment with romidepsin, tumor size and weight were obviously reduced compared with the control group (Fig. 2a). The expression of cleaved-caspase 3 in romidepsin-treated group was also upregulated (Fig. 2b).In the CAC model, colon cancer was induced by AOM and 3% DSS treatment for 70 days and then treated with romidepsin three times (Fig. 2c). The average number of tumors per mouse was used as criterion of efficacy, which was divided based on size (< 5 mm and ≥ 5 mm in the largest dimension). The average number of tumors ≥ 5 mm per mouse was lower in the romidepsin treatment group than in the control group, while there was no obvious difference in the number of tumors with sizes < 5 mm between the two groups (Fig. 2d). These results showed that romidepsin could inhibit colon cancer growth in vitro and in vivo by inhibiting cancer cell proliferation, inducing cell cycle arrest and promoting apoptosis.

### Romidepsin upregulated PD-L1 in mouse colon cancer cells

**Fig. 3:**
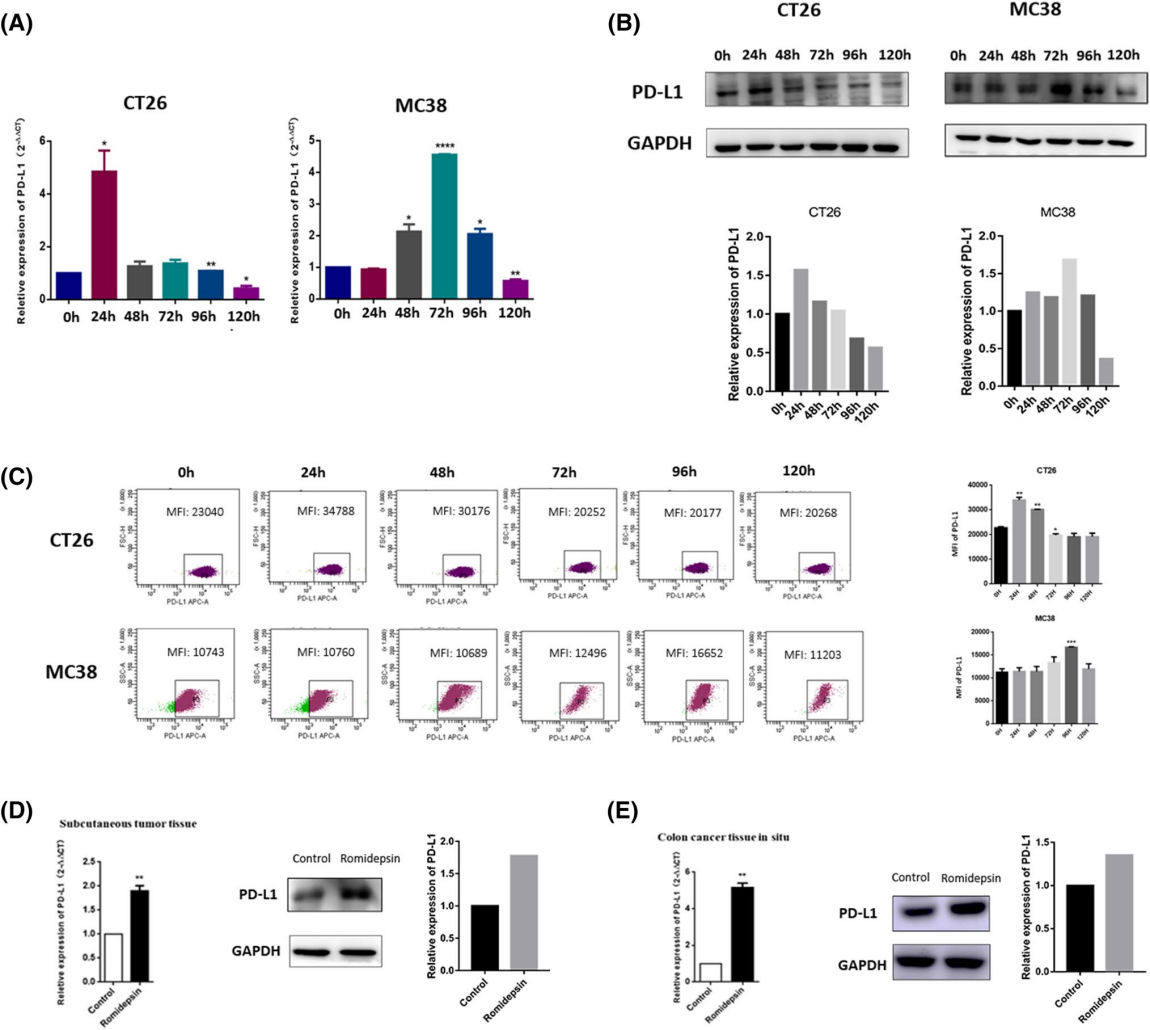
Romidepsin upregulation of PD-L1 expression in murine colon cancer cells. **a** The mRNA level of PD-L1 in CT26 and MC38 cells was detected through qRT-PCR after treated with romidepsin for 0, 24, 48, 72, 96 and 120 h. Each experiment was repeated three times. **b** The protein level of PD-L1 in colon cancer cells was assayed by western blot. **c** The expression of PD-L1 on the surface of colon cancer cells was analyzed by mean fluorescence intensity (MFI) using flow cytometry. **d** In subcutaneous transplantation tumor models, the mRNA levels of PD-L1 in control group (*n* = 5) and romidepsin group (*n* = 5) were determined by qRT-PCR, and *t*-test was used for statistics in the left. The protein levels of PD-L1 were determined by western blot in the middle and right. **e** In CAC models, the mRNA levels of PD-L1 in control group (*n* = 7) and romidepsin group (*n* = 7) were determined by qRT-PCR, and *t* test was used for statistics in the left. The protein levels of PD-L1 were determined by western blot in the middle and right (*P < 0.05, **P < 0.01, ***P < 0.001)

Our study indicated that romidepsin arrested colon cancer growth directly. We further analyzed its effects on PD-L1 expression in cancer cells and immune responses. Following treatment with romidepsin, the qRT-PCR and Western blotting assay results showed that the cells experienced PD-L1 upregulation at both the mRNA and protein levels (Fig. 3a, b). These findings were further confirmed by evaluating the mean fluorescence intensity (MFI) of PD-L1 using flow cytometry (Fig. 3c). The ability of romidepsin to upregulate PD-L1 in vivo was also investigated using a subcutaneous transplantation tumor model and a CAC model. After treatment with romidepsin, PD-L1 expression in the tumors was assessed by qRT-PCR and Western blotting. Consistent with the in vitro data, romidepsin treatment could significantly increase the PD-L1 expression in tumors (Fig. 3d, e).

### Romidepsin increased PD-L1 expression by regulating histone acetylation and BRD4

**Fig. 4:**
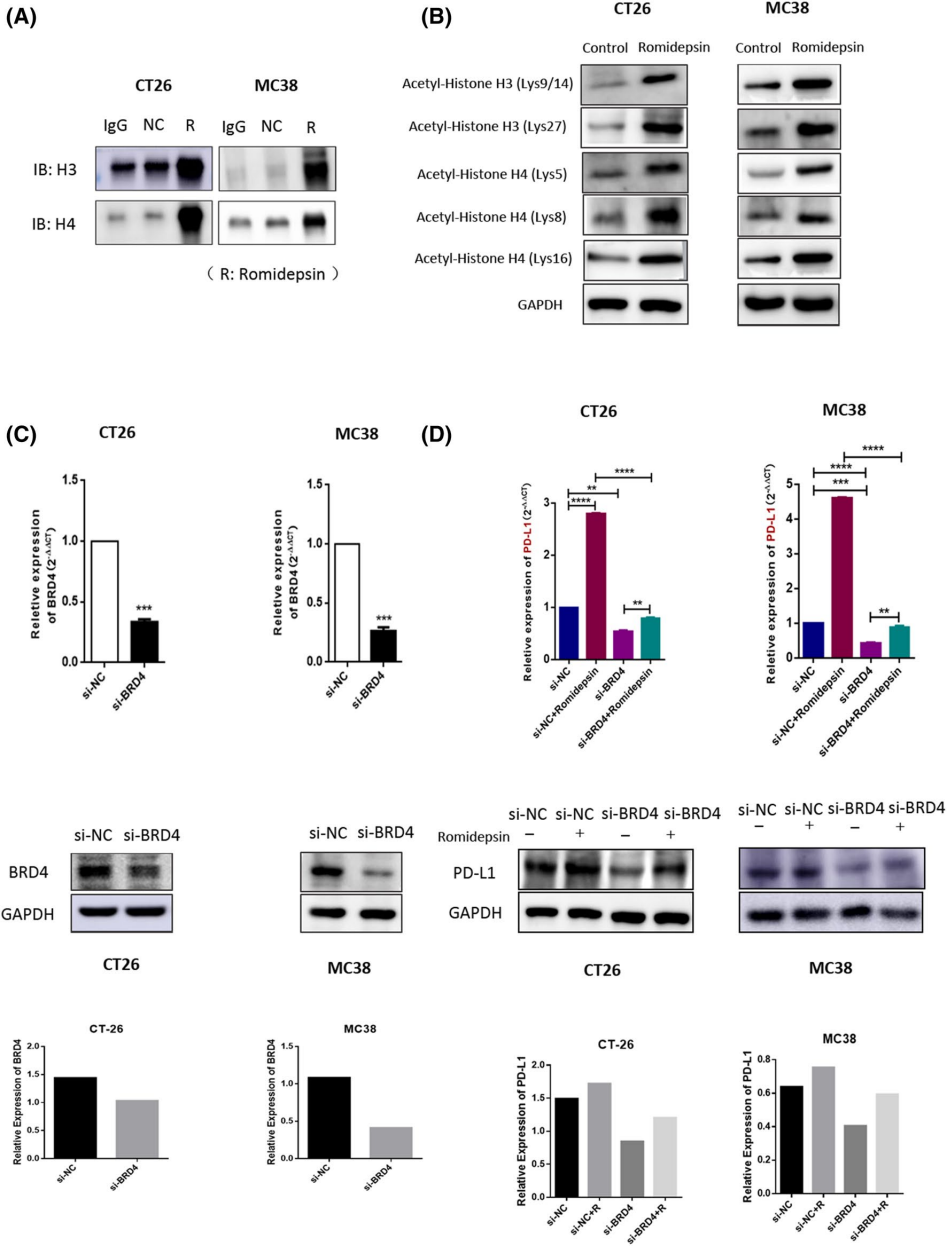
Romidepsin induced PD-L1 expression by regulating histone acetylation and BRD4. **a** CT26 and MC38 cells were treated with romidepsin for 24 h. Acetylated proteins in the cells were harvested by immunoprecipitation. Western blotting was used to measure the levels of histone H3/H4 acetylated proteins. **b** The acetylation levels of histone H4-Lys5, H4-Lys8, H4-Lys16, H3-Lys9/14 and H3-Lys27 sites were analyzed by western blot after treatment with control or romidepsin. **c** A pool of three siRNAs (100 nM in total) was introduced into CT26 and MC38 cells to knock down BRD4. The BRD4 expression in these cells was measured via RT-qPCR and western blot. The western blot signals had been quantified and relative expression level was showed. **d** CT26 and MC38 cells were treated with si-NC, si-NC + romidepsin, si-BRD4 and si-BRD4 + romidepsin, respectively. The PD-L1 expression in different groups was measured by qRT-PCR and western blot (*P < 0.05, **P < 0.01, ***P < 0.001)

Because romidepsin enhanced PD-L1 expression in colon cancer cells, we examined the possible mechanisms by which romidepsin regulated PD-L1 expression. The histone acetylation state and expression levels of BRD4, a transcription factor for PD-L1, were analyzed before and after treatment with romidepsin. After treatment with romidepsin, the acetylation of histone 3 and histone 4 at different lysine sites was increased (Fig. 4a, b). In addition to the acetylation of histones 3 and 4, we detected the role of bromodomain-containing proteins (BRD4) in PD-L1 expression upregulation by romidepsin. BRD4 plays a critical role in regulating the transcription of PD-L1, whose binding sites are acetylated lysines 5 and 8 of histone H4 (H4K5ac/K8ac). Pools of three siRNAs (100 nM in total) were used to inhibit BRD4 expression in CT26 and MC38 cells (Fig. 4c). The effects of upregulating PD-L1 via romidepsin in the BRD4 knockdown group were much less than those in the control group (Fig. 4d), which indicated that BRD4 participated in regulating PD-L1 via romidepsin.

### Romidepsin increased the prevalence of Tregs, but combination with an anti-PD-1 antibody reversed this effect

**Fig. 5:**
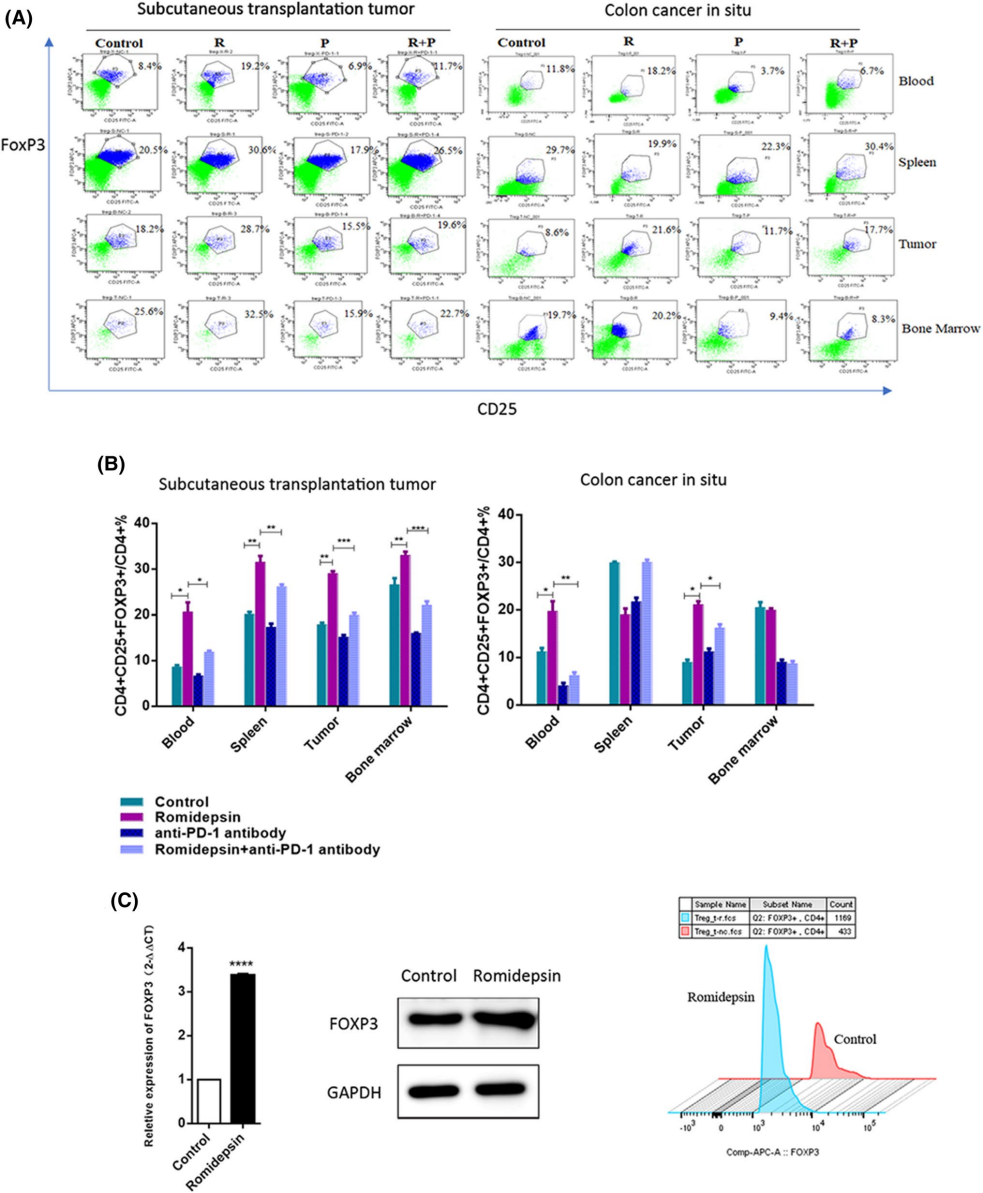
Romidepsin increased the prevalence of FOXP3 + Tregs in vivo*.* **a** Subcutaneous transplantation tumor mice and CAC mice were treated with romidepsin (R), anti-PD-1 antibody (P), combination treatment (R + P) or saline (control). Flow cytometry was conducted to detect Foxp3 + Tregs in the blood, spleen, tumor and bone marrow. **b** The percentage of CD4 + CD25 + Foxp3 + Tregs in CD4 + T cells after four treatments (control, romidepsin, anti-PD-1 antibody, romidepsin + anti-PD-1 antibody) was described. **c** CD4 + CD25 + T cells were sorted from the spleens of BABL/c mice by MACS and treated with romidepsin for 24 h. The expression level of Foxp3 in CD4 + CD25 + T cells was analyzed by qRT-PCR, western blot and flow cytometry (*P < 0.05, **P < 0.01, ***P < 0.001)

Because romidepsin increased the expression of PD-L1 in colon cancer cells, we next evaluated the influence of romidepsin on T cells, which was mediated by crosstalk between cancer cells and T cells through PD-L1/PD-1 signaling. Subcutaneous transplantation tumor models and CAC models were used to assess the effect. These mice were treated with romidepsin (R), anti-PD-1 antibody (P), combination treatment (R + P) or saline (control). The percentages of Foxp3 + Tregs in the blood, spleen, tumors and bone marrow were quantified by flow cytometry assays (Fig. 5a). Mice in the romidepsin group had a significantly higher percentage of Foxp3+ Tregs than the control group, and this effect was partially reversed when combined with the anti-PD-1 treatment (Fig. 5b). This phenomenon could be observed in subcutaneously transplanted tumor mice and in only the blood and tumors of CAC mice (Fig. 5b). Furthermore, to determine the role of romidepsin in Foxp3+ Treg regulation, we sorted CD4+ CD25+ T cells from the spleens of BABL/c mice by MACS. The expression of Foxp3 in CD4+ CD25+ T cells was significantly increased after treatment with romidepsin (Fig. 5c).

### Romidepsin decreased the Th1/Th2 ratio and IFN-γ secretion of CD8+ T cells, while combination with an anti-PD-1 antibody reversed these effects

**Fig. 6:**
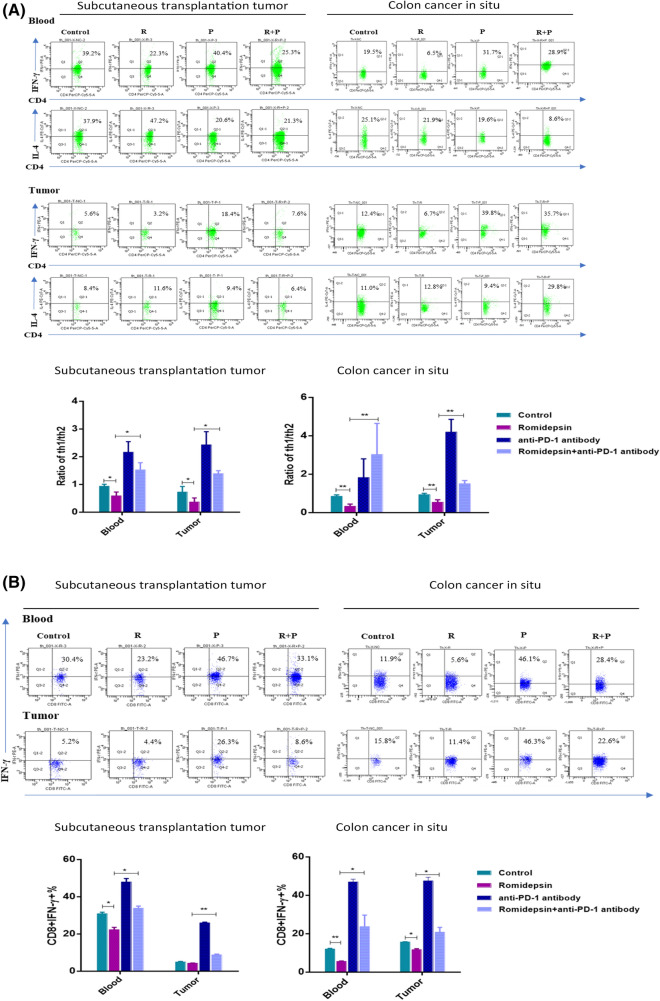
Romidepsin decreased the Th1/Th2 ratio and IFN- γ secretion by CD8 + T cells. **a** In blood and tumors of the subcutaneous transplantation tumor model and CAC model (5 mice in each group), the levels of IFN-γ and IL-4 in CD4 + T cells were detected by flow cytometry after treatment. The ratio of IFN-γ/IL-4 was analyzed, which could reveal the Th1/Th2 balance. **b** The state of IFN-γ + CD8 + T cells under different treatments was assayed by flow cytometry in the subcutaneous transplantation tumor model and CAC model (*P* < 0.05, **P < 0.01, ***P < 0.001, ****P < 0.0001)

Intracellular T-helper 1/T-helper 2 (Th1/Th2) cytokines, including IFN-γ and IL-4, were detected by flow cytometric assays. In blood and tumors of subcutaneously transplanted tumor mice and CAC mice, the Th1/Th2 ratio (shown by IFN-γ/IL-4) was decreased after romidepsin treatment, which could be reversed when combined with anti-PD-1 treatment (Fig. 6a). IFN-γ secretion was examined to evaluate the activation of CD8+ T cells. Flow cytometry showed that romidepsin decreased the prevalence of CD8+ IFN-γ+ T cells. When combined with the anti-PD-1 antibody, the percentage of CD8+ IFN-γ+ cells were increased than romidepsin treatment alone (Fig. 6b).

### Anti-tumor effects of romidepsin in combination with an anti-PD-1 antibody in vivo

**Fig. 7:**
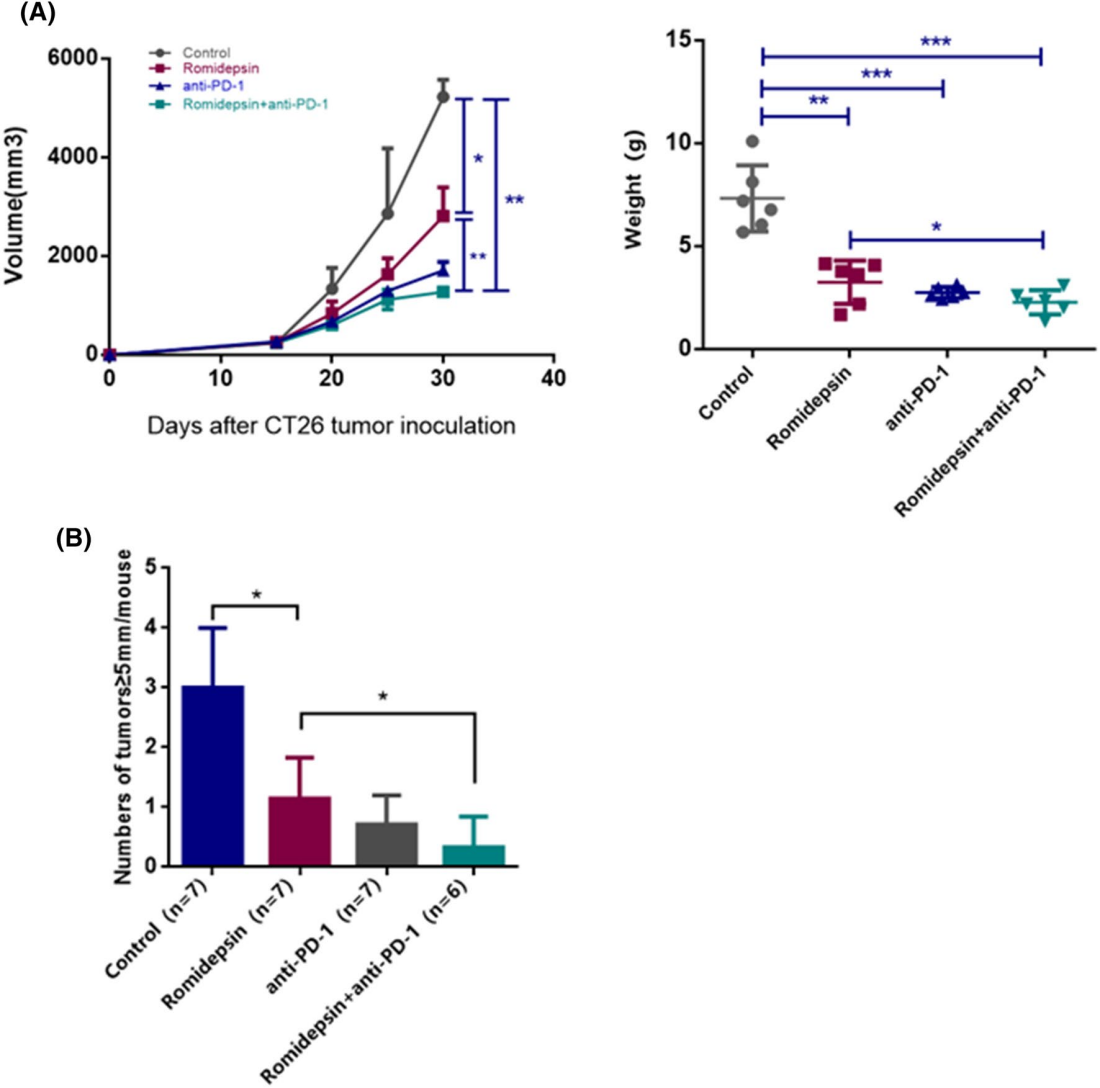
Anti-tumor effects of romidepsin in combination with an anti-PD-1 antibody. **a** The size and weight of tumors were analyzed as therapeutic indicators under the treatment of romidepsin, anti-PD-1 antibody and combination of two drugs. **b** Twenty-eight mice with colitis-associated cancer were randomly divided into four groups, which were treated with saline, romidepsin, anti-PD-1 antibody and romidepsin + anti-PD-1 antibody, respectively. After the treatment, the average number of tumors per mouse (≥ 5 mm in the largest dimension) was analyzed to evaluate efficacy (*P* < 0.05, **P < 0.01, ***P < 0.001, ****P < 0.0001)

The anti-tumor effects of romidepsin and anti-PD-1 antibody co-treatment were evaluated in subcutaneously transplanted tumor mice and in CAC mice. In subcutaneously transplanted tumor mice, the inhibitory effect of the co-treatment on the tumor growth was significantly increased compared with the treatment with romidepsin alone. And compared with anti-PD-1 antibody treatment, the tumor suppression effect of the combination therapy also showed an increasing trend (Fig. 7a). The incidences and multiplicity of colon neoplasms in CAC mice are shown in Fig. 7b. The average number of tumors ≥ 5 mm per mouse in the romidepsin combined with anti-PD-1 blockade treatment group tended to be less than that in the romidepsin treatment only group. In addition, compared with anti-PD-1 treatment, the group of combination also had an advantage in efficacy with no significant statistically difference.

## Discussion

Romidepsin is an HDACi involved in regulating cell proliferation, differentiation and apoptosis by promoting chromatin uncoiling, histone acetylation and genes transcription. Previous work with romidepsin indicated that it had considerable anti-tumor effects in solid tumors [18–20].

In our study, after being treated with romidepsin, the proportion of colon cancer cells in the G0/G1 phase increased significantly, while cells in the S + G2/M phase decreased. Romidepsin can induce cell cycle arrest and inhibit cell proliferation. In addition, romidepsin also promotes cell apoptosis, which was verified by changes of cleaved-caspase 3. Furthermore, romidepsin can inhibit tumor growth in vivo in both subcutaneous tumor-transplanted models and CAC models.

Beyond the direct suppression of tumor cells, romidepsin has been reported to have the ability to regulate the immune system [14, 21]. We found that PD-L1 was upregulated in colon cancer cells and tissues after being treated with romidepsin, which can be explained by two mechanisms. First, romidepsin increased the level of acetylation of histone H3/H4 in humor cells, which showed a positive correlation with the expression of PD-L1. The result is consistent with previous understanding of the regulation of PD-L1 expression, that is, HDACis enhance the expression of PD-L1 by increasing the acetylation of histone H3/H4 at the promoter region of PD-L1 genes [14]. In addition, we also found that the expression of PD-L1 was related to the transcription factor BRD4 after being treated with romidepsin. BRD4, a novel class of epigenetic “readers”, are involved in the long-term control of genome activity through their ability to bind with acetylated lysine residues in histones [22]. It has been reported that acetylated lysine 5 and 8 of nucleosome histone H4 (H4K5ac/K8ac), which were upregulated after romidepsin treatment, are BRD4 binding sites [23]. When BRD4 in CT26 and MC38 cells was knocked down, the effect of romidepsin on up-regulating PD-L1 expression was greatly reduced. Therefore, it is reasonable to speculate that romidepsin can upregulate the expression of PD-L1 by regulating BRD4 [24–26].

Although the mechanism of PD-L1 upregulation after romidepsin treatment were elucidated as above, the effect of this change on anti-tumor immune response has been controversial. PD-L1 is an important immunomodulatory molecule. Mechanistically, after the combination of PD-1 receptor on T cells and its ligand PD-L1, tyrosine phosphatases are recruited to dephosphorylate downstream effector molecules and thereby attenuate T cell receptor (TCR)-mediated signaling, which ultimately inhibits T cell proliferation and cytokine production [27, 28]. PD-1 blocking antibodies have the ability to inhibit the PD-L1/PD-1 pathway and restore T cell function. On the one hand, the expression of PD-L1 on tumor cells negatively regulates T cell responses and allows immune escape [29, 30]. On the other hand, increasing evidences show that HDACi-induced upregulation of PD-L1 can increase T cell infiltration, upregulate antigen presentation and thus enhance the efficacy of anti-PD-1 immunotherapy [21, 31, 32]. Our results indicate that the upregulation of PD-L1 expression induced by romidepsin treatment suppresses cellular immune functions in colon cancer, including downregulating the ratio of Th1/Th2 cells (shown as IFN-γ/IL-4) and the secretion of IFN-γ in CD8+ T cells, as well as upregulating the percentage of Foxp3+ Tregs. The effect of HDAC inhibitors on Tregs differentiation has been previously reported. HDAC inhibitors can promote the expression of Foxp3 and preserve Foxp3 lysine ɛ-acetylation, which will inhibit the ubiquitination degradation of Foxp3 and enhance its binding to DNA [16]. Foxp3 binds to DNA and regulates the transcription of multiple genes in Treg cells, thereby promoting Treg development and immunosuppressive function [15–17, 33]. In our research, romidepsin is likely to increase the percentage of Tregs by upregulating Foxp3 expression.

Even if romidepsin treatment produced a series of immunosuppression, the results of subcutaneous tumor-transplanted models and CAC models confirmed its inhibitory effect on tumor growth. And due to its immune relevance, the combined strategy of HDACis and anti-PD-1 immunotherapy was inspired. Compared with the treatment of romidepsin or anti-PD-1 antibodies alone, the combination of the two drugs not only downregulates the immunosuppression of romidepsin but also increases the tumor killing effect of anti-PD-1 immunotherapy, ultimately achieving the optimal anti-tumor effect. Therefore, the combination of romidepsin and anti-PD-1 antibody provides a more potential option for colon cancer treatment.

## Abbreviations

AOM: Azoxymethane
BRD4: Bromodomain-containing protein 4
CAC: Colitis-associated cancer
DSS: Dextran sodium sulfate
HDACi: Histone deacetylase inhibitor
HDACs: Histone deacetylases
MFI: Mean fluorescence intensity
PD-1: Programmed cell death 1
PD-L1: Programmed cell death-Ligand 1
TCR: T cell receptor
Tregs: Regulatory T cells
